# Characterization of the TBR1 interactome: variants associated with neurodevelopmental disorders disrupt novel protein interactions

**DOI:** 10.1093/hmg/ddac311

**Published:** 2022-12-29

**Authors:** Elliot Sollis, Joery den Hoed, Marti Quevedo, Sara B Estruch, Arianna Vino, Dick H W Dekkers, Jeroen A A Demmers, Raymond Poot, Pelagia Deriziotis, Simon E Fisher

**Affiliations:** Language and Genetics Department, Max Planck Institute for Psycholinguistics, Nijmegen, XD 6525, The Netherlands; Language and Genetics Department, Max Planck Institute for Psycholinguistics, Nijmegen, XD 6525, The Netherlands; Department of Cell Biology, Erasmus University Medical Center, Rotterdam, CN 3015, The Netherlands; Department of Plant Physiology, Umeå Plant Science Centre, Umeå, 90736, Sweden; Language and Genetics Department, Max Planck Institute for Psycholinguistics, Nijmegen, XD 6525, The Netherlands; Language and Genetics Department, Max Planck Institute for Psycholinguistics, Nijmegen, XD 6525, The Netherlands; Proteomics Center, Erasmus University Medical Center, Rotterdam, CN 3015, The Netherlands; Proteomics Center, Erasmus University Medical Center, Rotterdam, CN 3015, The Netherlands; Department of Cell Biology, Erasmus University Medical Center, Rotterdam, CN 3015, The Netherlands; Language and Genetics Department, Max Planck Institute for Psycholinguistics, Nijmegen, XD 6525, The Netherlands; Language and Genetics Department, Max Planck Institute for Psycholinguistics, Nijmegen, XD 6525, The Netherlands; Donders Institute for Brain, Cognition and Behaviour, Radboud University, Nijmegen, AJ 6525, The Netherlands

## Abstract

TBR1 is a neuron-specific transcription factor involved in brain development and implicated in a neurodevelopmental disorder (NDD) combining features of autism spectrum disorder (ASD), intellectual disability (ID) and speech delay. TBR1 has been previously shown to interact with a small number of transcription factors and co-factors also involved in NDDs (including CASK, FOXP1/2/4 and BCL11A), suggesting that the wider TBR1 interactome may have a significant bearing on normal and abnormal brain development. Here, we have identified approximately 250 putative TBR1-interaction partners by affinity purification coupled to mass spectrometry. As well as known TBR1-interactors such as CASK, the identified partners include transcription factors and chromatin modifiers, along with ASD- and ID-related proteins. Five interaction candidates were independently validated using bioluminescence resonance energy transfer assays. We went on to test the interaction of these candidates with TBR1 protein variants implicated in cases of NDD. The assays uncovered disturbed interactions for NDD-associated variants and identified two distinct protein-binding domains of TBR1 that have essential roles in protein–protein interaction.

## Introduction

Recurrent heterozygous disruptions of the human *TBR1* gene (including whole gene deletions, missense variants and truncating mutations) have been reported to cause neurodevelopmental disorders (NDDs), including autism spectrum disorder (ASD), intellectual disability (ID) and speech delay (OMIM 606053) (1–8).

*TBR1* expression is strongly enriched in the brain (9), where it is largely restricted to post-mitotic neurons (10). Studies in mice have revealed that *Tbr1* expression reaches its peak during embryogenesis and gradually decreases postnatally (10). Around birth, the gene is highly expressed in the cerebral cortex, primarily in layer 6, but also in layers 2/3 and in a minority of neurons in layer 5 (10,11). It is also expressed in embryonic amygdala (12,13), hippocampus (10,14), olfactory bulb (15) and deep cerebellar nuclei (16). TBR1 is a transcription factor (TF) and appears to play important regulatory roles in the development of many of these brain structures. In the mouse cortex, Tbr1 controls both regional and laminar neuronal identity, driving differentiation towards frontal cortex and layer-6 cell fates, while suppressing caudal and layer-5 identity (11,17,18). It also regulates transcriptional circuits related to dendritic spine and synapse formation in the cortex (19). In the amygdala, Tbr1 promotes cell migration, axonal outgrowth and the formation of inter- and intra-amygdalar connections (12,13).

At the molecular level, TBR1 binds to target DNA loci via its T-box DNA-binding domain and recognizes the T-box binding element AGGTGTGA (20). TBR1 appears to be able to function as either an activator or repressor of transcription. TBR1 binding sites identified by chromatin-immunoprecipitation (ChIPseq) screening are enriched for both active (H2K27ac, H3K4me1) and repressive (H3K9me3, H3K27me3) chromatin marks (21).

The regulatory functions of many TFs rely on interactions with other proteins, and TBR1 is no exception. TBR1 interacts with CASK, a membrane-associated guanylate kinase that is primarily expressed at neuronal synapses (22). This interaction allows CASK to enter the nucleus, where it acts as a coactivator with TBR1 to promote expression of TBR1 target genes, such as *RELN* and *GRIN2B* (22,23). TBR1 also interacts with the FOXP1/2/4 and BCL11A TFs (4,24), all of which have been associated with NDDs (25–28). The TBR1–FOXP2 interaction is abolished by pathogenic variants in either protein (4,24), and at least one of the known *FOXP1* pathogenic variants disrupts the TBR1–FOXP1 interaction (29).

It is likely that the interaction network of TBR1 is much more extensive than the proteins identified so far. Thus, in the current study, affinity purification-mass spectrometry (AP-MS) in human cell-lines was employed to characterize the TBR1 interactome. Candidate interactors that had also been implicated in NDDs were prioritized for independent validation using bioluminescence resonance energy transfer (BRET) assays. Follow-up experiments confirmed candidate interactions and demonstrated that known pathogenic *TBR1* variants can disrupt these novel interactions.

## Results

### AP-MS reveals novel TBR1-interaction candidates

**Figure 1:**
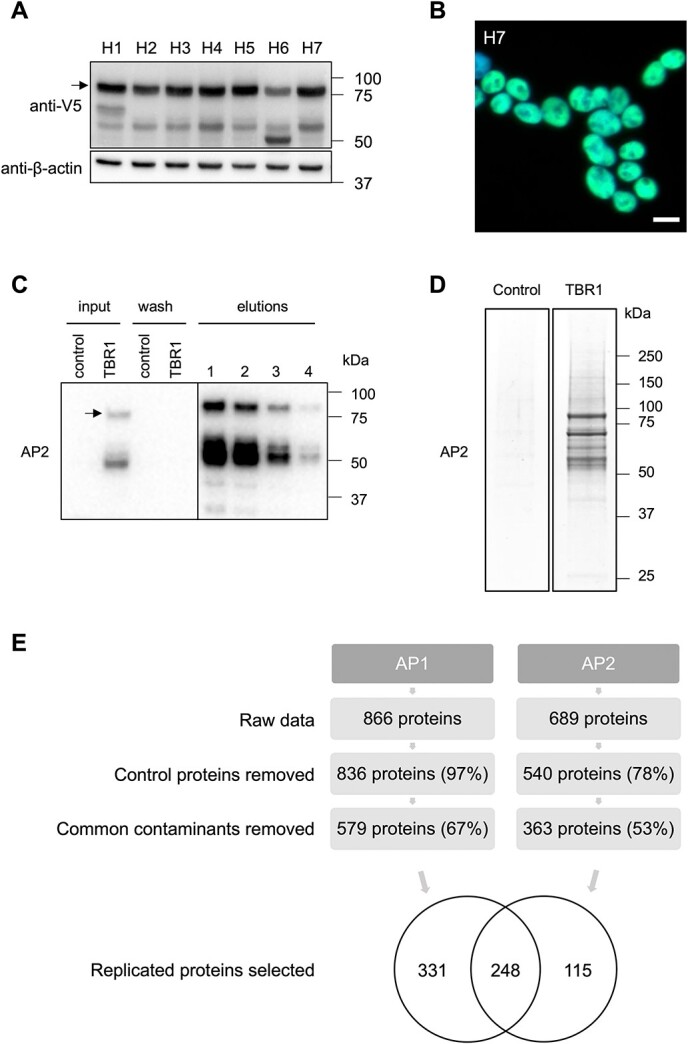
Affinity purification-mass spectrometry of TBR1 and bound proteins. (**A**) Immunoblotting of whole-cell lysate from HEK293 cells stably transfected with 2×FLAG/V5-TBR1, using anti-V5 antibody (1:3000). β-actin is also shown (1:10000). Arrow shows ~77.5 kDa band corresponding to TBR1. A second non-specific band was detected more faintly in all clones at ~60 kDa. As no alternative isoforms of TBR1 have been experimentally confirmed (https://www.uniprot.org/uniprot/Q16650) (63) this band is more likely due to degradation. Seven clones are shown, of which H7 was selected for affinity-purification experiments. (**B**) Immunofluorescence staining of nuclear TBR1 expression (green) in clone H7 using anti-V5 antibody (1:500) and Alexa 488 secondary antibody. Scale bar = 10 μm. (**C**) Representative affinity purification of TBR1-interacting proteins (AP2; AP1 shown in Supplementary Material, Fig. S1). Western blot shows total lysate (input) and washed proteins (wash) for empty HEK293 cells (control) and the TBR1-containing stable cell line (TBR1), and affinity-purified material (elutions 1–4; TBR1 stable cell line only). Immunoblotting performed with anti-FLAG primary antibody (1:1000) and HRP-conjugated anti-mouse secondary antibody (1:2000). Arrow shows ~77.5 kDa band corresponding to TBR1. (**D**) Coomassie-stained SDS-polyacrylamide gel of representative affinity purification of TBR1 and control cells (AP2; AP1 shown in Supplementary Material, Fig. S1). (**E**) Filtering of mass spectrometry results, showing the number of proteins (and percentage of the original) remaining from each experiment after removal of proteins detected in control cells and common contaminants. A total of 248 proteins detected in both experiments (43% of AP1 hits, 68% of AP2 hits) were carried through for further analysis.

Nuclear extraction and AP-MS were employed to identify novel TBR1-interaction candidates. Two independent affinity purifications (AP1 and AP2) were performed (Fig. 1; Supplementary Material, Fig. S1), with 248 proteins replicated in both experiments after quality control (Fig. 1E; Supplementary Material, Table S1). Relative protein abundance was assessed by exponentially modified protein abundance index (emPAI), an approximate measure that takes the size of the protein into account (30). Averaging across the two experiments, the most abundant proteins were TP53, STUB1, RAD50 and ZMYM4 (mean emPAI > 1).

Of the TBR1 interactors reported in prior literature, only CASK was identified in this AP-MS screen (mean emPAI = 0.20). The FOXP TFs were not detected, while BCL11A was detected in AP1 only, at relatively low levels (emPAI = 0.07). The absence of these proteins is unlikely to be explained by a lack of expression in HEK293 cells. RNA sequencing studies in HEK293 have detected moderate expression of FOXP1 (11.7 transcripts per million [TPM]), FOXP2 (6.7 TPM), FOXP4 (19.1 TPM) and BCL11A (12.2 TPM)—all higher than the median expression level for all genes in HEK293 (4.1 TPM) and within the same range as the expression of CASK (17.0 TPM) (9). These interactions might be more dependent on a specific cellular (e.g. neuronal) context, or might be relatively weak or transient and therefore disturbed by the cell lysis and washing steps in the AP.

### Cluster analysis identifies known transcriptional regulation complexes

**Figure 2:**
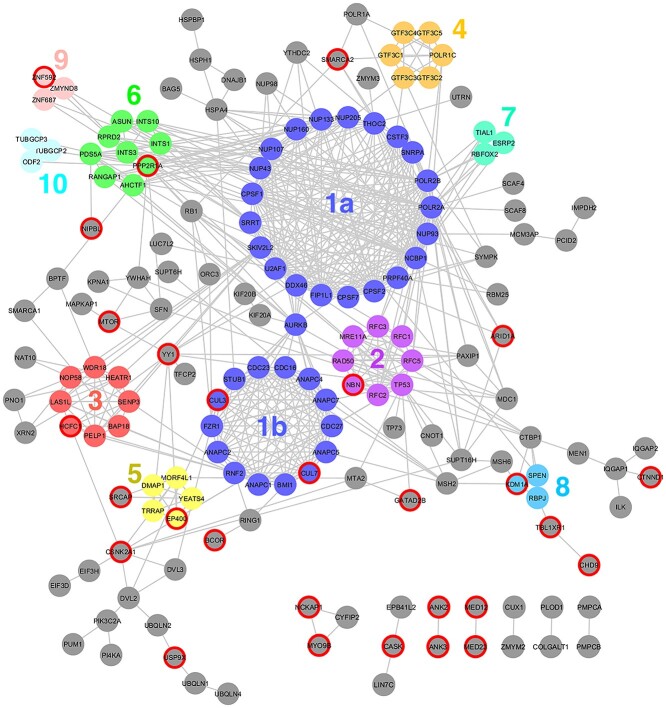
Graphical network depiction of the putative TBR1 interactome. Nodes represent proteins, connectors represent known interactions imported from the STRING database. The network comprises a large, connected component of 154 proteins (Component 1; top), 2 trios and 5 pairs (bottom right), and 78 isolated proteins (not shown). Highly interconnected clusters of proteins within Component 1, as identified by the MCODE algorithm, are grouped together and color coded. Proteins encoded by ASD/ID-related genes are marked with a red border.

The set of 248 putative TBR1 interactors was cross-referenced with the STRING database, to find known interactions within the network. There were 604 interactions amongst the 248 proteins, and each protein interacted with an average of 4.871 other proteins (Fig. 2). The most well-connected hubs were the RNA polymerase proteins POL2RA (34 interactions) and POLR2B (31 interactions), and the nuclear cap-binding protein NCBP1 (31 interactions). Other network statistics are summarized in Supplementary Material, Table S2.

The interactome was dominated by one large sub-network of 154 proteins (Component 1), alongside seven small clusters and 78 isolated proteins without established interactions. Within Component 1, a cluster analysis was performed using the MCODE algorithm (31) (Version 1.4.1) to identify highly interconnected regions likely to correspond to protein complexes or parts of pathways. Ten clusters were identified, comprising several known protein complexes and included proteins involved in RNA transcription, processing and export; chromatin modification; DNA replication and repair; cell cycle regulation and ubiquitination (Fig. 2; Supplementary Material, Table S3). The smaller clusters outside Component 1 had diverse functions and including actin-associated signaling proteins (NCKAP1-CYFIP2-MYO9B), two CASK interactors (LIN7C and EPB41L2), ankyrins (ANK2-ANK3), mediator complex members (MED12-MED23), collagen-modifying proteins (PLOD1-COLGALT1) and mitochondrial import proteins (PMPCA-PMPCB) (Fig. 2).

These results suggest that TBR1 interacts with multiple proteins to regulate transcription of protein-coding genes as well as non-coding RNAs, and that this TF activity is mediated by interactions with co-regulators and chromatin modifying complexes.

### Transcription factors and epigenetic factors in the TBR1 interactome

Our findings suggested two specific protein classes that might be important TBR1 interactors: (a) other TFs, and (b) epigenetic factors, including chromatin remodeling factors and histone modifiers.

Epigenetic factors (Supplementary Material, Table S4) were identified using the EpiFactors database, a manually curated database of epigenetic regulators, complexes and targets (32). Of the 248 putative TBR1-interactors, 52 (21%) were involved in histone modification and chromatin remodeling. These proteins are involved in a range of processes with both positive and negative effects on transcription, including histone (de)methylation, (de)acetylation, ubiquitination, deSUMOylation and nucleosome restructuring (Supplementary Material, Table S5). The diversity of interactions may help to explain the mixed activator/repressor functions that have been observed for TBR1 (11,17,21,33). The identified epigenetic factors included multiple members of Nu4A, MLL, SWI/SNF and Polycomb complexes, as noted above, as well as the NuRD histone deacetylation/chromatin remodeling complex.

The set of putative TBR1 interactors was also cross-referenced with a published list of human TFs (34) (Supplementary Material, Table S6). There were 26/248 (10%) confirmed or probable TFs amongst the TBR1-interacting proteins (Supplementary Material, Table S7). Note that six proteins (ADNP, TP53, YY1, ZBTB33, ZNF592 and ZNF687) were classified as both epigenetic factors and TFs.

### NDD-related proteins in the TBR1 interactome

Rare disruptive *TBR1* variants have been identified in patients with ASD and/or ID (1–3,5–8). Eleven (4%) of the putative TBR1-interactors are encoded by ASD candidate genes with at least suggestive evidence identified in the literature by expert curators (Supplementary Material, Tables S8 and S10) (35). There were also 24 (9%) putative interactors encoded by ID-related genes with a mutation identified in at least one ID patient (Supplementary Material, Tables S9 and S10). There was overlap between the two lists, with *ADNP, MTOR, POGZ* and *TBL1XR1* implicated both in ID syndromes and in ASD. In total therefore, the putative TBR1 interactome uncovered in this study included 31 ID/ASD-related proteins (Supplementary Material, Table S10), as defined by prior expert curation of the literature.

### BRET validation of novel TBR1-interacting proteins

Of the 248 putative TBR1 interaction partners identified by AP-MS, the 31 encoded by known ASD/ID-related genes (Supplementary Material, Table S10) were considered most likely to share a role with TBR1 in the etiology of NDDs. These proteins were ranked by emPAI (30), averaged across the two AP-MS experiments. Ten highly ranked candidates were selected for validation and further functional characterization: KDM1A, GATAD2B, NCKAP1, YY1, CSNK2A1, TBL1XR1, CTNND1, BCOR, ADNP and SMARCA2 (Supplementary Material, Fig. S2).

Since TBR1 is known to interact with three NDD-related TFs—FOXP1, FOXP2 and BCL11A (4,24)—we hypothesized that these proteins may have additional interactors in common with TBR1. Five putative TBR1-interactors were previously reported to interact with FOXP1/2: CTBP1, GATAD2B, NR2F2, YY1 and ZMYM2 (36–40). NR2F2 also interacts with BCL11A (41). Therefore, in addition to those listed as high-emPAI candidates above, three further candidates (CTBP1, NR2F2 and ZMYM2) were selected for follow-up (Supplementary Material, Fig S3). We also included CTBP2, which is 77% identical to CTBP1 at the amino-acid level and shares its interactions with FOXP1/2/4 (39); and NR2F1, which is 85% identical to NR2F2 and shares its interactions with FOXP1/2/4 (39) and BCL11A (41). Although these two candidates were each detected in only one of the two AP-MS experiments, they were considered promising candidates due to their similarity to CTBP1 and NR2F2 and their conserved interactions with other TFs.

**Figure 3:**
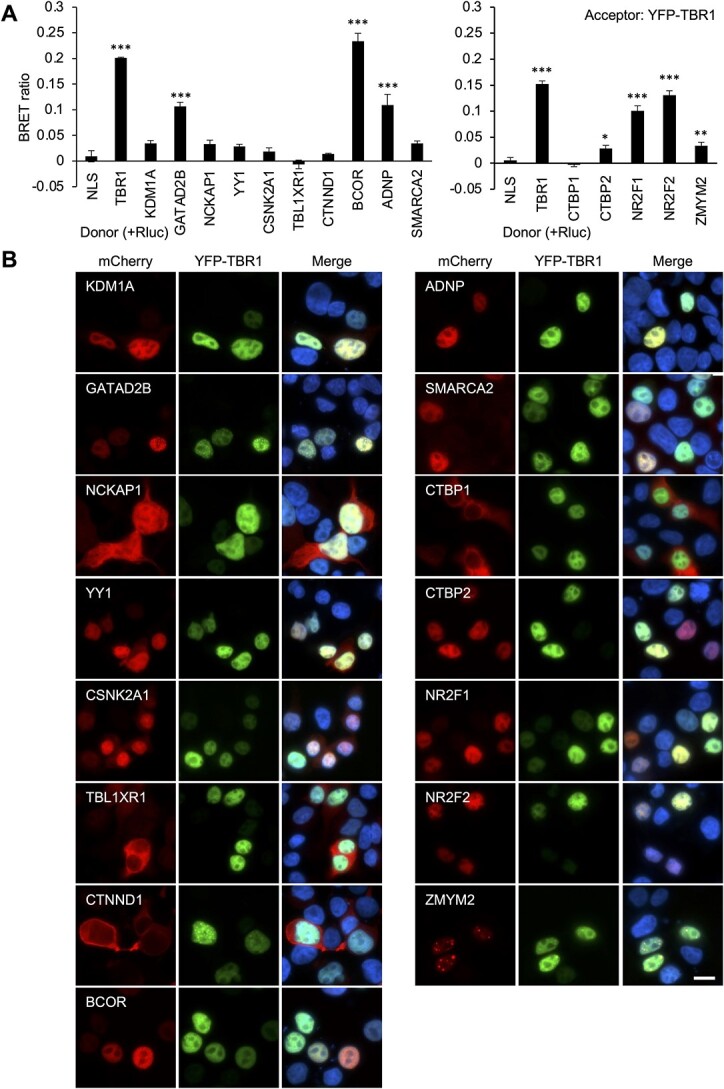
BRET validation of five novel TBR1-interactors: GATAD2B, BCOR, ADNP, NR2F1 and NR2F2. (**A**) BRET assays for interaction between TBR1 and 15 interaction candidates. Bars represent the corrected mean BRET ratios ±SD of one experiment performed in triplicate (^*^P < 0.05, ^*^^*^P < 0.01 and ^*^^*^^*^P < 0.001 compared to Rluc-NLS control, one-way ANOVA and post-hoc Tukey’s test). (**B**) Fluorescence microscopy images of HEK293 cells transfected with TBR1 (fused to YFP, green) and interaction candidates (fused to mCherry, red). Nuclei were stained with Hoechst 33342 (blue). Scale bar = 10 μm.

Interactions were validated using BRET assays, which offer an advantage over methods such as co-immunoprecipitation, by allowing detection of protein–protein interactions within live cells, and a greater scaling capacity to test multiple interactions in parallel (38). *Renilla* luciferase (Rluc)-fusion proteins were generated for the fifteen TBR1 interaction candidates and tested for interactions with YFP-TBR1 using BRET assays in HEK293 cells. Positive interactions (all *P* < 0.001) were detected between TBR1 and five of the candidates: GATAD2B, BCOR, ADNP, NR2F1 and NR2F2 (Fig. 3A). Significant BRET signals were also observed for CTBP2 (*P* = 0.016) and ZMYM2 (*P* = 0.003), which may indicate weak interactions with TBR1; however, the magnitude of these signals was considered too low to warrant further investigation here (Fig. 3A). Each of the confirmed interactors was expressed in the nucleus and co-localized with TBR1 in co-transfection experiments (Fig. 3B). Our BRET experiments did not detect interactions with the other eight candidates. TBL1XLR1, CTNND1 and CTBP1 remained predominantly cytoplasmic when co-transfected with TBR1, which could account for the observed lack of interaction (Fig. 3B). On the other hand, the remaining five candidates (KDM1A, NCKAP1, YY1, CSNK2A1 and SMARCA2) showed no evidence of interaction in BRET experiments (Fig. 3A), despite at least partial co-localization within the nucleus (Fig. 3B). While this might suggest false-positive results in the AP-MS, it is also possible that our BRET results included false-negatives, which can occur if proteins do interact, but the Rluc and YFP tags do not come into sufficiently close proximity for energy transfer to occur, due to mismatches in protein length, conformation or orientation (38).

### Interaction candidates are co-expressed with Tbr1 in the developing mouse brain

**Figure 4:**
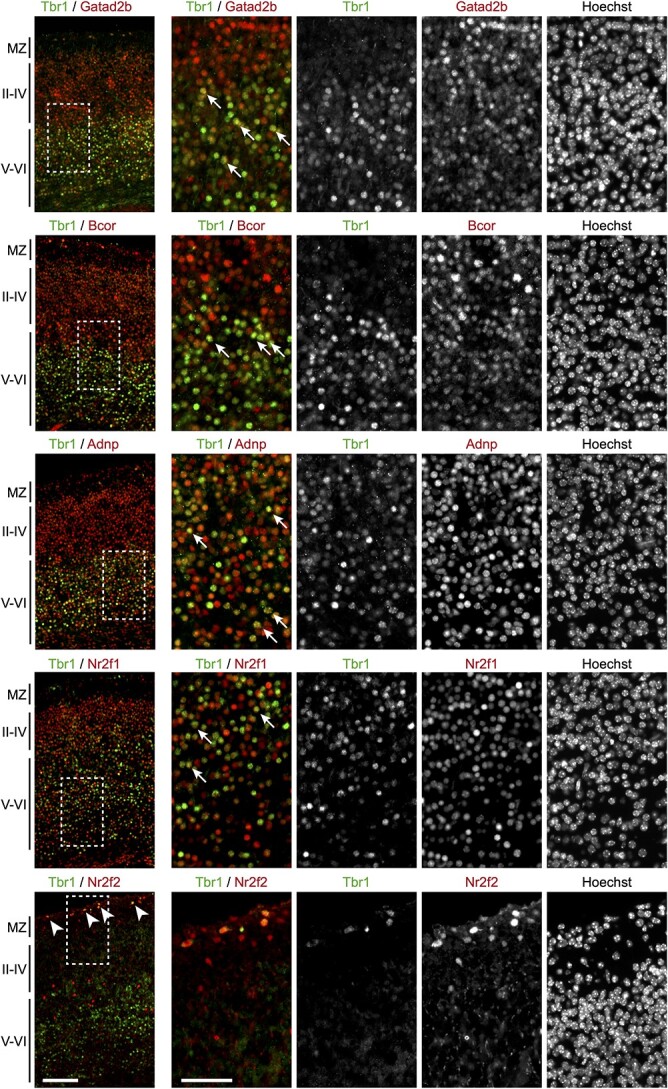
Tbr1 is co-expressed with Gatad2b, Bcor, Adnp, Nr2f1 and Nr2f2 in the cerebral cortex. Immunofluorescence experiments to assess endogenous co-expression of Gatad2b, Bcor, Adnp, Nr2f1 and Nr2f2 with Tbr1 in the cerebral cortex of E18.5 mouse embryos. Left, micrographs showing Tbr1 in green and its interactors in red. The marginal zone (MZ), upper cortical layers (II–IV) and deeper layers (V–VI) are indicated. The arrow heads show cells co-expressing Tbr1 and Nr2f2 in the MZ. Scale bar = 100 μm. Right, magnified views of the regions indicated with the dashed rectangles. Arrows indicate example cells with co-expression of Tbr1 and the interactor. Nuclei were stained with Hoechst 33342. Scale bar = 50 μm.

TBR1 expression is highly enriched in the brain (9) and largely restricted to neuronal cells (10), while our AP-MS experiment was performed in the HEK293 cell line. Although there is evidence that HEK293 cells originate from neural-related tissue (42,43), they are likely to differ considerably from TBR1-expressing neurons *in vivo*. To confirm the potential for interaction *in vivo*, we investigated co-expression of Tbr1 with its interactors in embryonic (E18.5) mouse cortical sections (Fig. 4). Gatad2b, Bcor, Adnp and Nr2f1 showed nuclear localization, with expression throughout the cortex. While the distribution of these interaction partners did not completely overlap with that of Tbr1, we observed co-expression in a subset of cells in the deeper layers (Fig. 4, indicated with arrows). Nr2f2 expression was restricted to the marginal zone and a sub-population of cells in the deeper layers, with co-expression with Tbr1 limited to cells in the marginal zone (Fig. 4, indicated with arrow heads). These may be Cajal–Retzius cells, where Tbr1 expression has been previously described (44).

We also looked up the broader list of 248 putative TBR1 interactors in single-cell transcriptomics data from the Human Protein Atlas (9), to confirm the potential to interact in the human brain. TBR1 itself was predominantly found in excitatory neurons, and 222/248 putative interactors (90%) were also detected (≥1 transcript per million) in that cell type.

### TBR1 variants disrupt novel interactions

Previous studies (4,24) have thoroughly characterized 11 *TBR1* variants identified in patients with NDDs (Supplementary Material, Table S11)—including their effects on subcellular localization, transcriptional repression and interactions with other proteins (Supplementary Material, Table S12). These include seven *de novo* variants (five missense, one nonsense and one frameshift) that are considered clinically pathogenic (1,4,45,46), and four variants of uncertain significance inherited from unaffected parents (4,47). In the current study, we used BRET assays to investigate how these variants might affect interactions between TBR1 and the five novel validated interactors. The patient variants, as well as two synthetic truncations (p.N394^*^, p.S568^*^), were also employed to map the GATAD2B-, ADNP-, BCOR- and NR2F1/2-binding sites and compare these to previously described protein-binding regions of TBR1.

**Figure 5:**
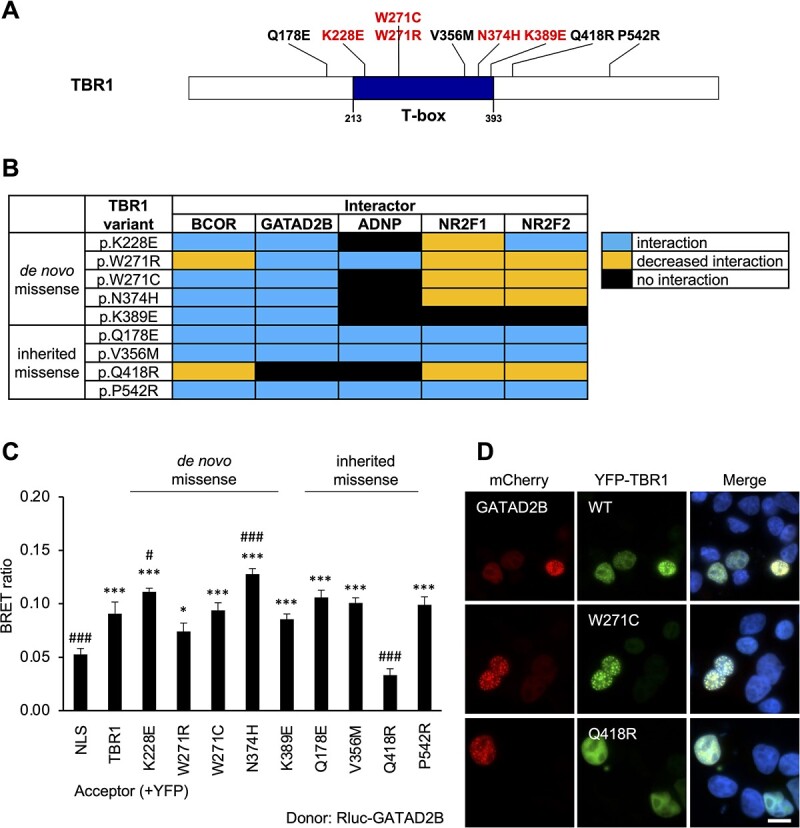
*TBR1* missense variants disrupt multiple novel interactions. (**A**) Protein diagram showing TBR1 missense variants tested in this study. The *de novo* variants (red) are concentrated within the T-box DNA-binding domain, while the rare inherited variants (black) are distributed throughout the protein. (**B**) Effects of TBR1 missense variants on interactions with the five novel TBR1-interactors. (**C**) Example BRET results for interaction between TBR1 missense variants and GATAD2B. Bars represent the corrected mean BRET ratios ±SD of one experiment performed in triplicate (^*^P < 0.05 and ^*^^*^^*^P < 0.001 compared to YFP-NLS control, #*P* < 0.05 and ###*P* < 0.001 compared to WT TBR1, one-way ANOVA and post-hoc Tukey’s test). (**D**) Example fluorescence microscopy images of HEK293 cells co-transfected with GATAD2B (fused to mCherry, red) and selected TBR1 variants (fused to YFP, green). Nuclei were stained with Hoechst 33342 (blue). Scale bar = 10 μm.

Five *de novo* missense variants cluster within the T-box domain of TBR1, while four rare inherited missense variants have a wider distribution along the protein (Fig. 5A). A subset of these missense variants impaired all five novel TBR1-interactions, but the precise pattern differed among the interactors (Fig. 5B). All five *de novo* missense variants in TBR1 (p.K228E, p.W271C, p.W271R, p.N374H and p.K389E) retained interaction with GATAD2B (Fig. 5C; Supplementary Material, Fig. S4), as well as BCOR (Supplementary Material, Fig. S5), with the caveat that the interaction between p.W271R and BCOR showed a significantly decreased signal compared to WT TBR1 and BCOR. These findings aligned with the pattern of effects previously seen for CASK- and BCL11A-interaction, as well as TBR1 homodimerization (Supplementary Material, Table S12) (4,24). In contrast, four *de novo* missense variants (p.K228E, p.W271C, p.N374H and p.K389E) abolished the interaction with ADNP (Supplementary Material, Fig. S6). The same variants were previously reported to disrupt TBR1-FOXP2 interaction (Supplementary Material, Table S12) (4). Four variants (p.K228E, p.W271C, p.W271R and p.N374H) exhibited a slightly reduced interaction signal with NR2F1 and NR2F2 (although this reduction was not significant for the interaction between p.K228E and NR2F2), while only p.K389E abolished these interactions (Supplementary Material, Figs S7 and S8). Although p.W271R and p.W271C affect the same residue, their effects on protein-interactions are not always concordant. These differences in functional effects have been discussed elsewhere (24) and might reflect the different physicochemical properties of the amino acids introduced in each case.

Of the rare inherited variants, only p.Q418R had any effect on the tested interactions (Fig. 5B), abolishing interaction with GATAD2B (Fig. 5C; Supplementary Material, Fig. S4) and ADNP (Supplementary Material, Fig. S6), and giving a reduced interaction with BCOR (Supplementary Material, Fig. S5), NR2F1 (Supplementary Material, Fig. S7) and NR2F2 (Supplementary Material, Fig. S8). This p.Q418R variant was previously shown to abolish interaction with FOXP2 and BCL11A, but not CASK or WT TBR1 (Supplementary Material, Table S12) (4,24). The present results lend further support for a pathogenic role for p.Q418R in NDDs, through the disruption of multiple protein–protein interactions. The other three rare inherited variants of TBR1 (p.Q178E, p.V356M and p.P542R) interacted with all TBR1-interactors in this and previous studies (Supplementary Material, Table S12) (4,24), suggesting that these variants are likely to be benign.

BRET results were supported by microscopy in cells co-transfected with TBR1 variants and interactors (Supplementary Material, Figs S4–S8). In most cases, the expression patterns of TBR1 variants and interactors did not differ significantly depending on the co-expressed protein partner. However, in co-expression with BCOR and GATAD2B, a speckled nuclear pattern was observed for some TBR1 variants that exhibit a diffuse pattern when transfected alone. In several cases, this aligned with their effects on interaction. Three rare inherited variants (p.Q178E, p.V356M and p.P542R) co-localized in speckles with both BCOR and GATAD2B, and interacted with both; while p.Q418R exhibited speckles only with BCOR (Supplementary Material, Fig. S5), with which it weakly interacted, but remained diffuse when co-expressed with GATAD2B (Fig. 5D; Supplementary Material, Fig. S4), with which it did not interact. A similar pattern was observed for one *de novo* missense variant (p.W271R), which appeared speckled when co-expressed with BCOR (Supplementary Material, Fig. S5) but not GATAD2B (Supplementary Material, Fig. S4), though this did not correspond to a difference in interaction.

**Figure 6:**
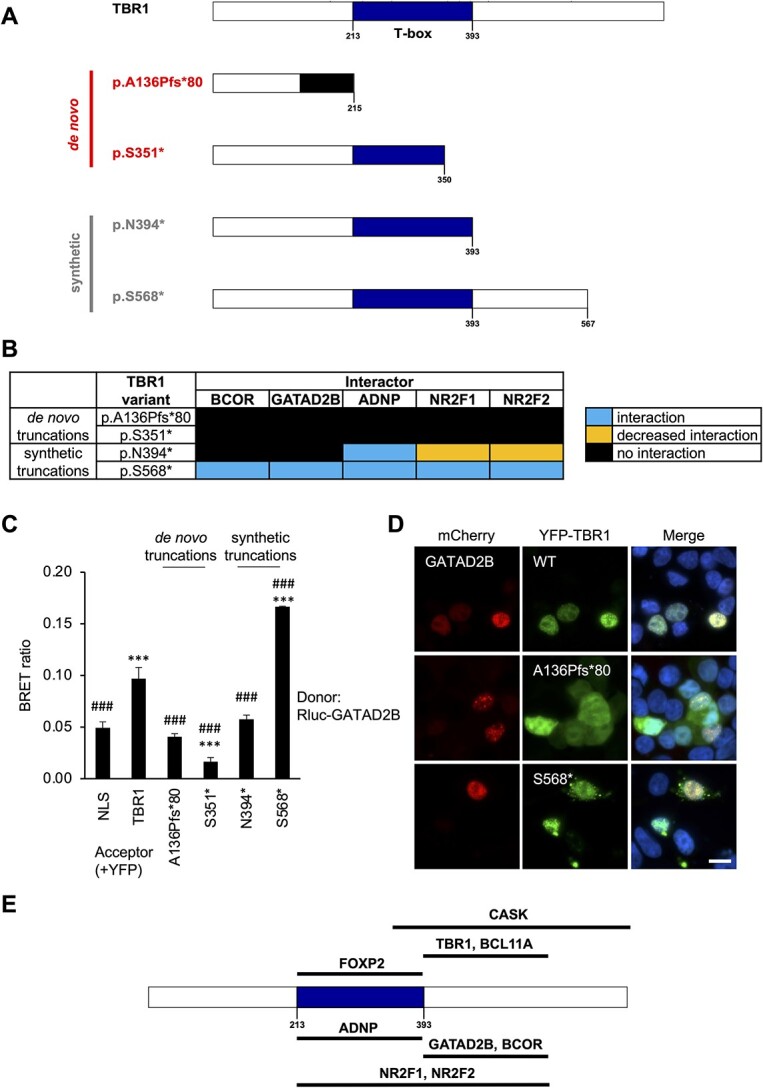
Mapping of proposed protein-binding regions on TBR1 using *TBR1* truncating variants. (**A**) Schematic representations of TBR1 truncating variants used in this study. Truncations are either *de novo* patient mutations (red) or synthetic constructs (grey). (**B**) Effects of TBR1 truncating variants on interactions with the five novel TBR1-interactors. (**C**) Example BRET results for interaction between TBR1 truncations and GATAD2B. Bars represent the corrected mean BRET ratios ±SD of one experiment performed in triplicate (^*^^*^^*^P < 0.001 compared to YFP-NLS control, ###*P* < 0.001 compared to WT TBR1, one-way ANOVA and post-hoc Tukey’s test). (**D**) Example fluorescence microscopy images of HEK293 cells co-transfected with GATAD2B (fused to mCherry, red) and selected TBR1 truncations (fused to YFP, green). Nuclei were stained with Hoechst 33342 (blue). Scale bar = 10 μm. (**E**) Proposed binding regions on the TBR1 protein for previously reported (above) and novel (below) interaction partners. References for previously-reported binding regions: TBR1-homodimerization and FOXP2 (4), CASK (22), BCL11A (24).

As the patient-derived TBR1 truncations p.A136Pfs^*^80 and p.S351^*^ are predicted to lead to nonsense-mediated decay and remain unexpressed *in vivo*, they were tested here primarily for the purpose of mapping binding sites, alongside two synthetic truncations, p.N394^*^ and p.S568^*^, which truncate the protein at the end of the T-box, and within the C-terminal region, respectively (Fig. 6A). The *de novo* truncations abolished all five novel interactions (Fig. 6B; Supplementary Material, Figs S4–S8), as they did for all other known TBR1-interactions in prior studies (Supplementary Material, Table S12). The synthetic p.N394^*^ variant abolished interaction with GATAD2B (Fig. 6C; Supplementary Material, Fig. S4) and BCOR (Supplementary Material, Fig. S5) and gave a reduced interaction signal for NR2F1 (Supplementary Material, Fig. S7) and NR2F2 (Supplementary Material, Fig. S8), similar to results previously observed for WT TBR1 and BCL11A (Supplementary Material, Table S12) (4,24). Interaction with ADNP, on the other hand, was unaffected by p.N394^*^ (Supplementary Material, Fig. S6), resembling previous results for FOXP2 (Supplementary Material, Table S12) (4). Finally, the synthetic p.S568^*^ variant of TBR1 retained interaction with all novel and previously described interaction partners (Fig. 6B and C; Supplementary Material, Figs S4–S8 and Table S12). These interaction results did not appear to be closely related to localization, with all TBR1 truncations showing mixed nuclear and cytoplasmic expression when co-transfected with the interactors (Fig. 6D; Supplementary Material, Figs S4–S8).

Overall, the results from analyses of truncated versions of the protein indicate that a C-terminal region of TBR1 (residues 394–568) is required for interaction with BCOR and GATAD2B (Fig. 6E). Identical or overlapping regions have been identified as important for binding to CASK (residues 342–682) (22) and to BCL11A (residues 394–568) (24), as well as TBR1 homodimerization (residues 394–568) (4). The p.Q418R variant of TBR1 that abolishes multiple interactions also lies within this region, and may disrupt the binding site. ADNP- and FOXP2-interactions tolerate the loss of this region (Supplementary Material, Fig. S6) (4). However, they too are vulnerable to the p.Q418R variant, suggesting that it perhaps causes conformational changes that affect the structure of the protein as a whole, rather than simply blocking a specific binding surface. On the other hand, TBR1 interactions with ADNP, like FOXP2, appear to be dependent on the T-box domain (residues 213–393 of TBR1; Fig. 6E) and these are the only interactions entirely abolished by multiple missense variants in that region. Interactions of TBR1 with NR2F1/2 may involve both the T-box and C-terminal region of TBR1 (Fig. 6E), and a downstream segment of the T-box encompassing residue K389 might be particularly important for these interactions.

## Discussion

Through AP-MS screening, we replicated the previously published interaction between TBR1 and CASK (22), and identified 247 novel TBR1-interaction candidates. These included proteins involved in ASD and ID, aligning with the neurodevelopmental deficits seen in TBR1-related disorder (OMIM 606053). Interactors also included multiple members of chromatin remodeling complexes such as NuRD, CoREST, Nu4A and SWI/SNF, playing either repression or activation roles. These findings support results of ChIP-seq experiments, where TBR1-binding sites were enriched for both active (H3K27ac and H3K4me1) and repressive (H3K9me3 and H3K27me3) chromatin marks (21), as well as evidence of both up- and down-regulation of Tbr1 target genes in mouse neurons (11,17,33).

Of the 15 interaction candidates selected for further validation, five—GATAD2B, ADNP, BCOR, NR2F1 and NR2F2—were confirmed as novel TBR1-interacting proteins using BRET as an additional independent method. All five are expressed in the brain, and their mouse orthologs co-localize with Tbr1 in the developing cortex (Fig. 4). Previous studies report expression in additional regions where TBR1 is also expressed, such as the olfactory region, hippocampus and amygdala (9,48–53), where they may also plausibly interact *in vivo*. Developmental disorders caused by mutations in *GATAD2B* (OMIM 615074) (54), *ADNP* (OMIM 615873) (55), *BCOR* (OMIM 300166) (56) and *NR2F1* (OMIM 615722) (57) have features that overlap with those of *TBR1-*related disorder, including developmental delay, ID, speech and language impairments and autistic behaviors. While *NR2F2* mutations mainly cause congenital cardiac abnormalities (OMIM 615779) (58), developmental delay is also seen in some patients (59). Thus, interactions between these proteins and TBR1 may be important in shared pathways relevant to NDDs. Indeed, pathogenic *TBR1* variants found in cases of NDD had a deleterious impact on multiple interactions. Nonsense/frameshift variants, which truncate both the T-box and C-terminal region, were the most severe, abolishing all interactions tested so far (Fig. 6B; Supplementary Material, Table S12), while missense variants had variable effects, dependent on the interactor (Fig. 5B; Supplementary Material, Table S12). While our AP-MS screen and subsequent validations have been based on overexpressed TBR1, further studies in cellular models of relevant cell types, such as primary or stem cell-derived neuronal cultures or *in vivo* models, could further explore the relevance and roles of these novel TBR1 interactions based on endogenous proteins.

Our investigations have expanded the current knowledge on the protein-interaction domains of TBR1, identifying two main regions of interest (Fig. 6E). Interaction with ADNP, like FOXP2 (4), primarily involves the T-box domain (residues 213–393) and is disrupted by *de novo* missense variants within that region. The C-terminal region of TBR1 (residues 394–567) appears to be the major binding site for BCOR and GATAD2B. Notably, the T-box was not sufficient even for a partial interaction with BCOR and GATAD2B, unlike WT TBR1 and BCL11A. On the other hand, interactions with NR2F1 and NR2F2 were affected both by missense variants in the T-box and by the p.N394^*^ truncation, indicating the involvement of both the T-box and C-terminal regions. It is interesting to note that the interaction partners that are most frequently affected by T-box mutations—FOXP2, ADNP, and to a lesser extent, NR2F1/2—are DNA-binding TFs, while those that are least affected—BCL11A, BCOR, CASK and GATAD2B—are co-factors that do not have direct DNA-binding domains. Perhaps interactions between TBR1 and other TFs require both proteins to be bound to DNA, while interactions between TBR1 and non-TF proteins are DNA-independent.

Recently, another study of the protein–protein interactions of 109 human transcription factors, which included a proximity-dependent biotinylation (BioID) screen for TBR1 interactors, identified 76 TBR1-interacting proteins (60). Seven proteins overlapped between our study and theirs, representing ~9% of proteins identified in the BioID study and ~3% of proteins identified in our AP-MS experiments. These include BCOR, which was validated here, but also SMARCA2, which did not validate in our BRET assays. The other overlapping proteins were ARID1A, CHD7, PAXIP1, ZFHX4 and ZMYM4, which are therefore strong candidates for future investigations. The BioID study also identified FOXP4, an established TBR1-interactor (4) that was absent from our results. The modest overlap of proteins identified in the two studies likely reflects methodological differences between AP-MS and BioID, the latter being potentially more efficient for studying transient interactions (60).

In conclusion, the work presented here substantially expands the known TBR1 interactome with the confirmation of five novel interactors. These include TFs and chromatin modifiers involved in both positive and negative regulation of transcription, supporting dual roles for TBR1 in regulating gene expression. Pathogenic *TBR1* variants can disrupt all five interactions, though the precise set of interactions affected by each variant differs, suggesting multiple potential etiological mechanisms for *TBR1*-related NDDs.

## Materials and Methods

### DNA constructs

*TBR1* was synthesized by GenScript USA, as previously described (4). *CTBP1, CTBP2, YY1, NR2F1, NR2F2, ZMYM2* were cloned as previously described (38–40). *KDM1A, GATAD2B, NCKAP1, CSNK2A1, TBL1XR1, CTNND1, BCOR, ADNP* and *SMARCA2* were amplified by PCR from human fetal cDNA (see Supplementary Material, Table S13 for primer sequences). Open reading frames of these genes were subcloned into pLuc, pYFP and a modified pmCherry-C1 expression vector (Clontech). Generation of constructs with *TBR1* variants has been described previously (4,24).

For generating stable TBR1-expressing cell lines for APs, the coding sequence of TBR1 was amplified from a plasmid template with BglII and XhoI restriction sites and inserted with N-terminal double-FLAG and V5 tags into a puromycin-resistant pPyCAG vector (61). TBR1 forward primer (5′ to 3′, BglII site underlined): agatctcagctggagcactgcctttc. Reverse primer (5′ to 3′, XhoI site underlined): ctcgagctagctgtgcgagtagaagc. All constructs were verified by Sanger sequencing.

### Cell culture and stable transfection

Rapidly proliferating HEK293 cells were used, to maximize the yield of input material for AP-MS. Cells were cultured in DMEM supplemented with 10% fetal bovine serum. As HEK293 cells do not endogenously express TBR1 (9), stable cell lines were generated as follows. The pPyCAG-2×FLAG/V5-TBR1 plasmid was linearized by AdhI digestion and transfected into HEK293 cells using GeneJuice (Merck-Millipore), according to the manufacturer's instructions. Seven clones were isolated following selection with culture medium containing 10 μm puromycin and maintained in culture medium containing 5 μm puromycin. The expression of tagged TBR1 protein in selected clones was confirmed using an anti-V5 antibody (Abcam, ab27671), by Western blotting (1:3000; Fig. 1A) and by immunofluorescence (1:500; Fig. 1B). Selected stable cell lines were maintained in culture medium containing 5 μm puromycin. One clone (H7) was selected for the AP-MS experiments.

### Nuclear extraction and FLAG-TBR1 affinity purification

The 2×FLAG/V5-TBR1 construct was localized to the nucleus (Fig. 1B), in agreement with the typical TBR1 expression pattern reported in the literature (4). For this reason, a nuclear extraction step was included in the AP-MS protocol, to maximize the concentration and therefore optimize the detection of TBR1 and its interaction partners, which were also hypothesized to be predominantly nuclear. HEK293 cells stably expressing 2×FLAG/V5-TBR1, and untransfected control cells, were expanded to confluence in twenty 15 cm dishes, harvested by scraping in PBS and nuclear extracts were prepared following Dignam *et al*. (61). Two separate nuclear extracts were prepared for each condition, and APs were performed in duplicate.

The AP procedure has been described previously (62). Briefly, nuclear extracts were dialyzed into buffer C-100 (20 mm HEPES pH 7.6, 20% glycerol, 100 mm KCl, 1.5 mm MgCl_2_, 0.2 mm EDTA) and 1.5 ml nuclear extract incubated with anti-FLAG M2 agarose beads (Sigma) for 3 h at 4°C. Nuclear extract was supplemented with 225 units of benzonase (Novagen) to digest DNA and 50 μg/ml ethidium bromide to inhibit DNA-protein associations (63). Beads were washed five times for 5 min with buffer C-100 containing 0.02% NP-40 (C-100^*^) and bound proteins eluted four times for 15 min at 4°C with buffer C-100^*^ containing 0.2 mg/ml FLAG-tripeptide (Sigma). Elution of proteins was validated by Western blot (Fig. 1C; Supplementary Material, Fig. S1A), and the first two elutions were pooled for each condition. Proteins were TCA precipitated and separated by polyacrylamide gel electrophoresis (Fig. 1D; Supplementary Material, Fig. S1B).

### Mass spectrometry

Mass spectrometry was performed by the Proteomics Centre at the Erasmus University Medical Center, as previously described (62). Briefly, 1D SDS-PAGE gel lanes were prepared by in-gel reduction with dithiothreitol, alkylation with iodoacetamide and digestion with trypsin. Nanoflow LC–MS/MS was performed on an 1100 series capillary LC system (Agilent Technologies) connected to an LTQ-Orbitrap mass spectrometer (Thermo). Mass spectra were acquired and searched against the UniProt human proteome database (UP000005640, accessed February 2016) (64) using the Mascot search algorithm (version 2.5.2). Each protein identification was assigned a Mascot score, equal to −10^*^log_10_(*P*), where *P* is the probability that the observed match is a random event. Peptides with a Mascot score lower than 40 (i.e. *P* > 10^−4^) were excluded. An emPAI score was also calculated for each protein hit, which incorporates the number of peptides identified per protein normalized by the theoretical number of peptides for that protein (30). This score corrects for the fact that, for the same number of molecules, proteins of greater size or with many peptides in the preferred mass range for mass spectrometry will generate more observed peptides.

### Filtering

Preliminary data preparation was done using Microsoft Excel and R. Filtering was performed in Cytoscape (version 3.5.0) (Fig. 1E). Contaminants, including human keratins, bovine serum proteins introduced during cell culture, and trypsin used for protein fragmentation, were removed from each list. For each experiment, non-specific hits were removed by retaining only those proteins detected in the TBR1-expressing cells and not in control cells. Protein hits were then filtered further by removing common background contaminants obtained from the Contaminant Repository for Affinity Purification (CRAPome) database (accessed April 2017) (65): data were retrieved for 30 control experiments matched for similar experimental conditions to the present study (HEK293, FLAG-tag, agarose beads), and if a protein occurred in more than 1 of these control experiments, it was excluded from the putative TBR1 interactome as a probable contaminant. Only proteins that were replicated in two independent AP-MS experiments were selected for inclusion in the final list of confident interaction partners (Supplementary Material, Table S1).

### Network analysis

Network analysis of the putative TBR1 interactome was performed in Cytoscape (version 3.5.0). Known interactions within the network were imported from the STRING database (version 10.5) (66), with a minimum required interaction score of 0.700 (high confidence) and allowing only interactions supported by experimental evidence or curated databases. The MCODE (Molecular Complex Detection) algorithm (version 1.4.1) (31) was used to identify highly interconnected regions within the network.

### Protein annotation

Proteins were annotated as TFs according to a curated list of human sequence-specific DNA-binding TFs (34). We considered all proteins defined by the authors as probable TFs (classes 'a', 'b' or 'other') or as possible TFs that contain InterPro domains that are only ever found in TFs (class 'c') (total *n* = 1493; Supplementary Material, Table S6) (34). Epigenetic factor status (*n* = 719 proteins; Supplementary Material, Table S4) and complex membership were assigned according to the Epifactors database (accessed May 2017) (32). ASD candidate genes (*n* = 190; Supplementary Material, Table S8) were taken from the Simons Foundation Autism Research Initiative database for ASD (SFARI Gene 2.0, accessed May 2017) (35). Genes with a SFARI score in category (1) High Confidence, (2) Strong Candidate or (3) Suggestive Evidence were included, while lower confidence categories were excluded. Genes related to syndromic forms of ASD were included. ID candidate genes (*n* = 748; Supplementary Material, Table S9) with a mutation identified in at least one patient were taken from the Radboud University Human Genetics Department diagnostic sequencing panel (version DG2.5; see http://www.radboudumc.nl/en/patientenzorg/onderzoeken/exome-sequencing-diagnostics/exomepanelspreviousversions/intellectual-disability).

### Fluorescence microscopy

Cells were seeded onto coverslips coated with poly-l-lysine (Sigma-Aldrich) and were fixed 24 h post-transfection using 4% paraformaldehyde (Electron Microscopy Sciences) for 10 min at room temperature. YFP and mCherry fusion proteins were visualized by direct fluorescence. Nuclei were visualized with Hoechst 33342 (Invitrogen). Fluorescence images were obtained using an Axio Imager M2 upright microscope (Zeiss).

### Bioluminescence resonance energy transfer

Bioluminescence resonance energy transfer (BRET) assays were performed as previously described (4,38). In summary, cells were transfected with pairs of proteins with N-terminal Rluc and YFP tags, in 96-well plates. Rluc and YFP were used as control proteins (with a C-terminal nuclear localization signal [NLS]). EnduRen luciferase substrate (Promega) was added to cells 48 h after transfection at a final concentration of 60 μm and incubated for 4 h. Emission measurements were taken with a TECAN F200PRO microplate reader using the Blue1 and Green1 filters. After subtracting background readings taken from untransfected cells, corrected BRET ratios were calculated as follows: [Green1_(experimental condition)_/Blue1_(experimental condition)_—Green1_(control condition)_/Blue1_(control condition)_], where the control condition represents cells transfected with Rluc-NLS alone.

### Immunohistochemistry

Mouse embryos (C57BL/6J; Charles River) were harvested at day E18.5 and fixed in 4% PFA (Electron Microscopy Sciences) overnight at 4°C. Brains were dissected out, placed in 30% sucrose overnight at 4°C and embedded in Tissue Tek OCT Compound (Sakura). Cryosections of 8 μm were prepared on a Leica CM1950. For the rabbit-anti-TBR1 (1:500, ab31940; Abcam), goat-anti-BCOR (1:100, ab5276; Abcam), goat-anti-ADNP (1:100, PA5-47792; ThermoFisher) and goat-anti-GATAD2B (1:250, ab111248; Abcam) antibodies, antigen retrieval was performed using citrate buffer (pH 6.0; Sigma) at 65°C for 20 min. Afterwards, sections were blocked and permeabilized in blocking buffer (PBS with 5% donkey serum (Sigma) and 0.25% Triton-X100) for 1 h at room temperature. Primary antibodies were applied in blocking buffer overnight at 4°C. Secondary antibodies AlexaFluor 488 donkey-anti-goat and AlexaFluor 594 donkey-anti-rabbit were incubated in blocking buffer (both 1:500, Invitrogen) for 2 h at room temperature. For the chicken-anti-TBR1 (1:100, AB2261, Millipore), rabbit-anti-NR2F1 (1:250, ab181137; Abcam) and rabbit-anti-NR2F2 (1:250, ab42672; Abcam) antibodies, sections were post-fixed in 4% PFA for 10 min at room temperature. Afterwards antigen retrieval was performed using citrate buffer (pH 6.0; Sigma) at 65°C for 20 min, and sections were blocked and permeabilized in blocking buffer (1% BSA (Sigma) and 0.25% Triton-X100) for 30 min at room temperature. Primary antibodies were applied in blocking buffer for 72 h at 4°C. Secondary antibodies AlexaFluor 488 donkey-anti-chicken (1:250, Jackson ImmunoResearch) and AlexaFluor 594 donkey-anti-rabbit (1:500, Invitrogen) were incubated in blocking buffer for 3 h at room temperature. All sections were stained with Hoechst 33342 followed by Sudan Black B (Sigma) staining and then mounted in Fluorescent Mounting Medium (Dako). Images were acquired using the Zeiss AxioScan.Z1.

## Supplementary Material

HMG-2022-CE-00467_R1-Sollis-SuppFigs_ddac311

HMG-2022-CE-00467_R1-Sollis-SuppTables_ddac311
