# Supplementary material for: Characterization of the TBR1 interactome: variants associated with neurodevelopmental disorders disrupt novel protein interactions
Source: Hum Mol Genet. 2022 Dec 29;32(9):1497–510. doi: 10.1093/hmg/ddac311 (PMC10117376; doi:10.1093/hmg/ddac311)
Supplement: HMG-2022-CE-00467_R1-Sollis-SuppFigs_ddac311 [file hmg-2022-ce-00467_r1-sollis-suppfigs_ddac311.zip › HMG-2022-CE-00467.R1-Sollis-SuppFigs_ddac311.pdf]

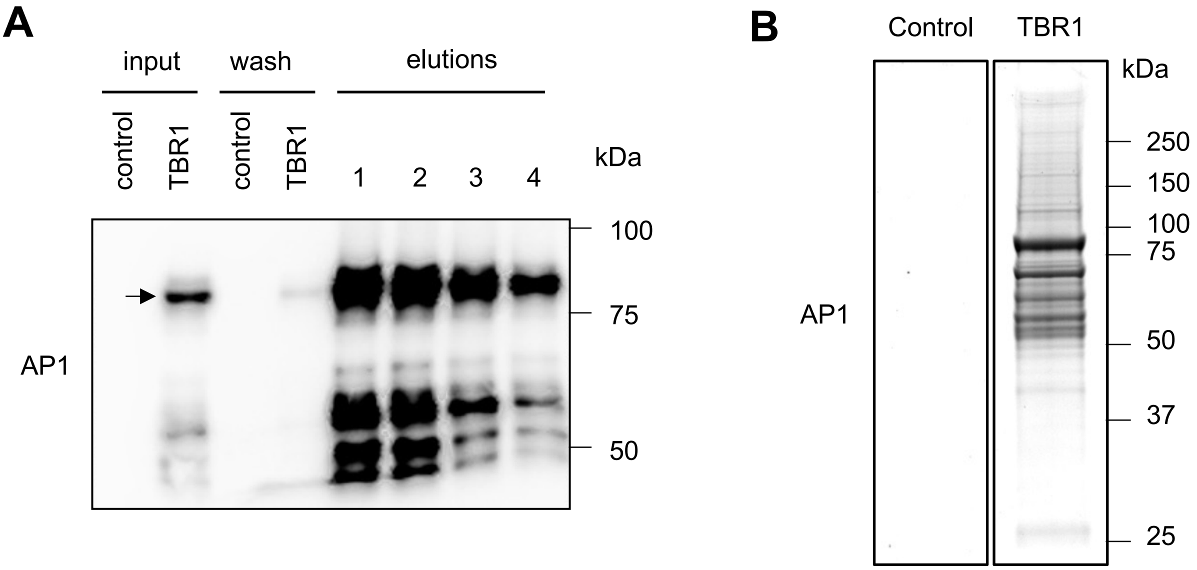

Figure S1. Affinity purification of TBR1-interacting proteins (AP1). (**A**) Western blot shows total lysate (input) and washed proteins (wash) for empty HEK293 cells (control) and the TBR1-containing stable cell line (TBR1), and affinity-purified material (elutions 1-4; TBR1 stable cell line only). Immunoblotting performed with anti-FLAG primary antibody (1:1000) and HRP-conjugated anti- mouse secondary antibody (1:2,000). Arrow shows ~77.5kDa band corresponding to TBR1. (**B**) Coomassie-stained SDS-polyacrylamide gel of affinity purification of TBR1 and control cells (AP1).

A

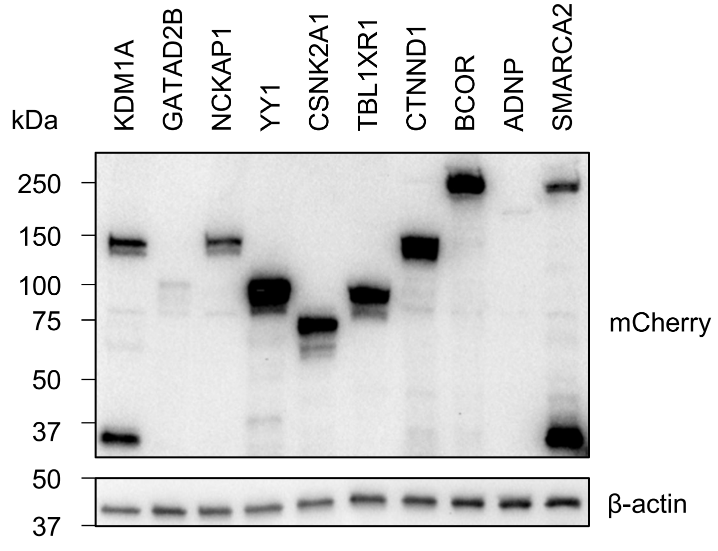

	MW (kDa)	
	Protein	+mCherry
KDM1A	92.9	121.7
GATAD2B	65.3	94.1
NCKAP1	128.8	157.6
YY1	44.7	73.5
CSNK2A1	45.1	73.9
TBL1XR1	55.6	84.4
CTNND1	105.0	133.8
BCOR	192.2	221.0
ADNP	122.8	151.6
SMARCA2	181.3	210.1

B

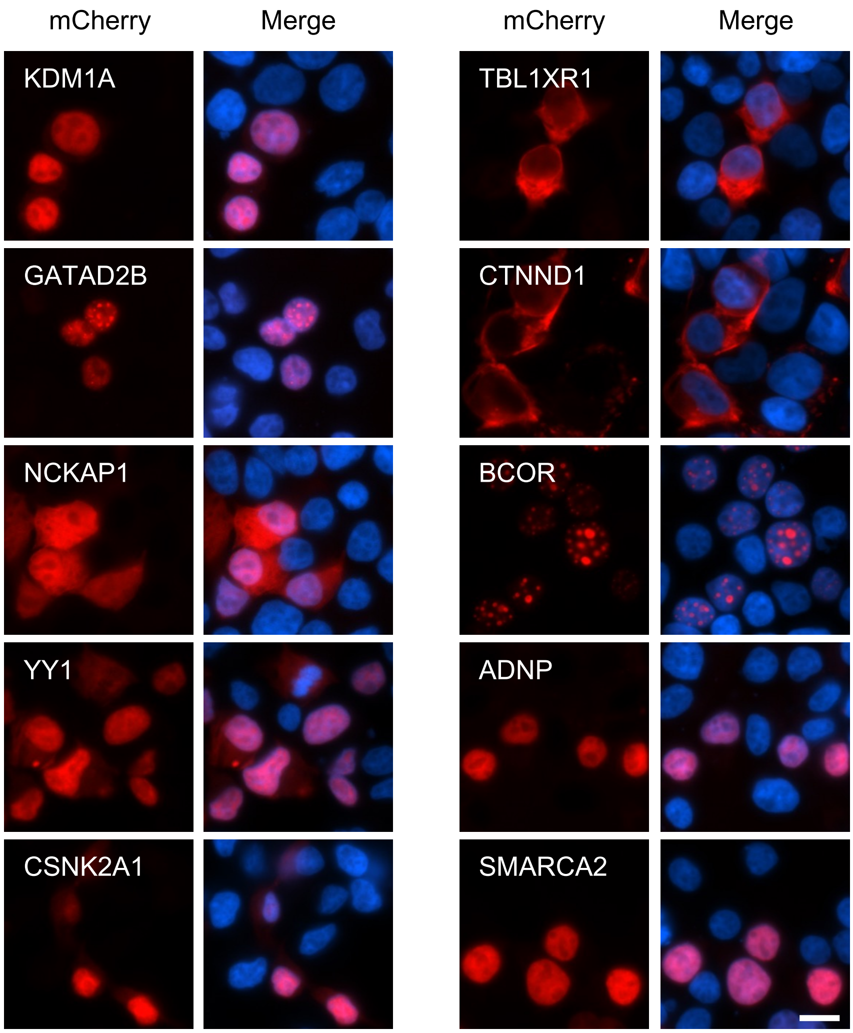

Figure S2. Expression of highly-ranked TBR1 interaction candidates. Candidates shown in this figure were selected on the basis of high mean emPAI scores across the two AP experiments. **(A)** Left panel: Immunoblotting of whole-cell lysates from HEK293 cells transfected with TBR1 interaction candidates fused to mCherry. β-actin served as a loading control. Right panel: expected molecular weights of candidates alone and as mCherry fusion proteins. **(B)** Fluorescence microscopy images of HEK293 cells transfected with TBR1 interaction candidates (fused to mCherry, red). Nuclei were stained with Hoechst 33342 (blue). Scale bar = 10 µm.

A

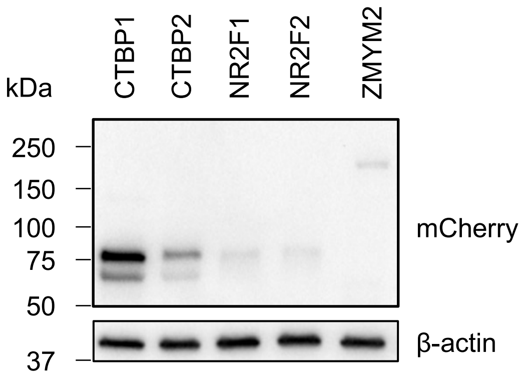

	MW (kDa)	
	**Protein**	**+mCherry**
CTBP1	47.5	76.3
CTBP2	48.9	77.7
NR2F1	46.2	75.0
NR2F2	45.6	74.4
ZMYM2	154.9	183.7

B

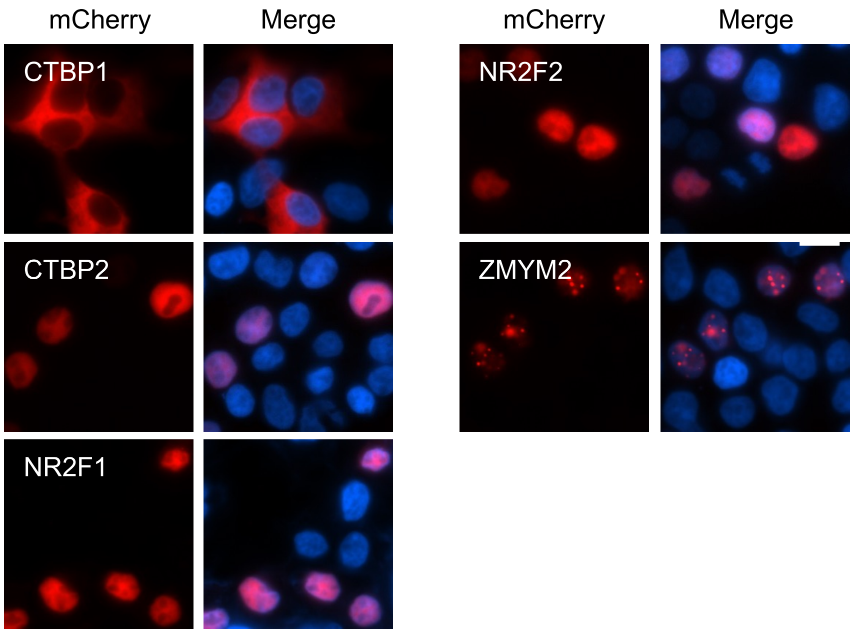

Figure S3. Expression of TBR1 interaction candidates with connections to known TBR1-interactors. Candidates shown in this figure were selected because they, or close homologs, have been reported to interact with the known TBR1-interactors FOXP1/2 or BCL11A. **(A)** Left panel: Immunoblotting of whole-cell lysates from HEK293 cells transfected with TBR1 interaction candidates fused to mCherry. β-actin served as a loading control. Right panel: expected molecular weights of candidates alone and as mCherry fusion proteins. **(B)** Fluorescence microscopy images of HEK293 cells transfected with TBR1 interaction candidates (fused to mCherry, red). Nuclei were stained with Hoechst 33342 (blue). Scale bar = 10 µm.

Figure S4. Effects of *TBR1* variants on interaction with GATAD2B. (A) BRET assays for interaction between TBR1 variants and GATAD2B. Bars represent the corrected mean BRET ratios ±SD of one experiment performed in triplicate (*P < 0.05 and ***P < 0.001 compared to YFP-NLS control, #P < 0.05 and ###P < 0.001 compared to WT TBR1, one-way ANOVA and *post-hoc* Tukey's test). (**B**) Fluorescence microscopy images of HEK293 cells co-transfected with GATAD2B (fused to mCherry, red) and TBR1 variants (fused to YFP, green). Nuclei were stained with Hoechst 33342 (blue). Scale bar = 10 μm.

Figure S5. Effects of TBR1 variants on interaction with BCOR. (A) BRET assays for interaction between TBR1 variants and BCOR. Bars represent the corrected mean BRET ratios ±SD of one experiment performed in triplicate (***P < 0.001 compared to YFP-NLS control, #P < 0.05 and ###P < 0.001 compared to WT TBR1, one-way ANOVA and post-hoc Tukey's test). (**B**) Fluorescence microscopy images of HEK293 cells co-transfected with BCOR (fused to mCherry, red) and TBR1 variants (fused to YFP, green). Nuclei were stained with Hoechst 33342 (blue). Scale bar = 10 μm.

Figure S6. Effects of TBR1 variants on interaction with ADNP. (A) BRET assays for interaction between TBR1 variants and ADNP. Bars represent the corrected mean BRET ratios ±SD of one experiment performed in triplicate (*P < 0.05, **P < 0.01 and ***P < 0.001 compared to YFP-NLS control, #P < 0.05 and ###P < 0.001 compared to WT TBR1, one-way ANOVA and post-hoc Tukey's test). (**B**) Fluorescence microscopy images of HEK293 cells co-transfected with ADNP (fused to mCherry, red) and TBR1 variants (fused to YFP, green). Nuclei were stained with Hoechst 33342 (blue). Scale bar = 10 μm.

Figure S7. Effects of TBR1 variants on interaction with NR2F1. (A) BRET assays for interaction between TBR1 missense (left panel) and truncating (right panel) variants and NR2F1. Bars represent the corrected mean BRET ratios ±SD of one experiment performed in triplicate (*P < 0.05 and ***P < 0.001 compared to YFP-NLS control, #P < 0.05, ##P < 0.01 and ###P < 0.001 compared to WT TBR1, one-way ANOVA and post-hoc Tukey's test). (**B**) Fluorescence microscopy images of HEK293 cells co-transfected with NR2F1 (fused to mCherry, red) and TBR1 variants (fused to YFP, green). Nuclei were stained with Hoechst 33342 (blue). Scale bar = 10 μm.

Figure S8. Effects of TBR1 variants on interaction with NR2F2. (A) BRET assays for interaction between TBR1 variants and NR2F2. Bars represent the corrected mean BRET ratios ±SD of one experiment performed in triplicate (***P < 0.001 compared to YFP-NLS control, ###P < 0.001 compared to WT TBR1, one-way ANOVA and post-hoc Tukey's test). (**B**) Fluorescence microscopy images of HEK293 cells co-transfected with NR2F2 (fused to mCherry, red) and TBR1 variants (fused to YFP, green). Nuclei were stained with Hoechst 33342 (blue). Scale bar = 10 μm.

Figure S4

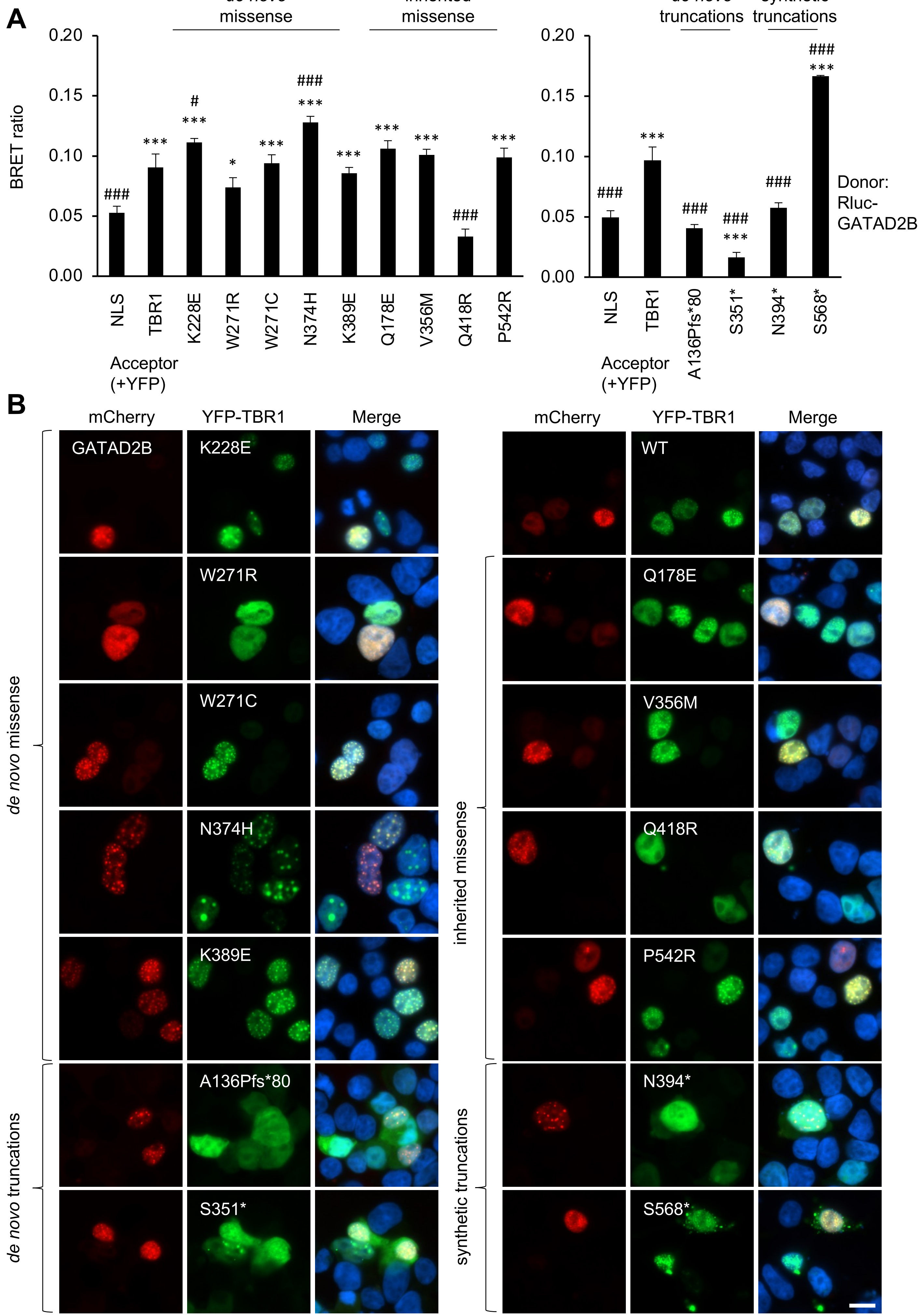

A
de novo missense
inherited missense
de novo truncations
synthetic truncations
BRET ratio
0.20
0.15
0.10
0.05
0.00
NLS
TBR1
K228E
W271R
W271C
N374H
K389E
Q178E
V356M
Q418R
P542R
A136Pfs*80
S351*
N394*
S568*
Acceptor (+YFP)
Donor: Rluc-GATAD2B
###
***
#
*
B
mCherry
YFP-TBR1
Merge
GATAD2B
WT
de novo missense
inherited missense
de novo truncations
synthetic truncations

Figure S5

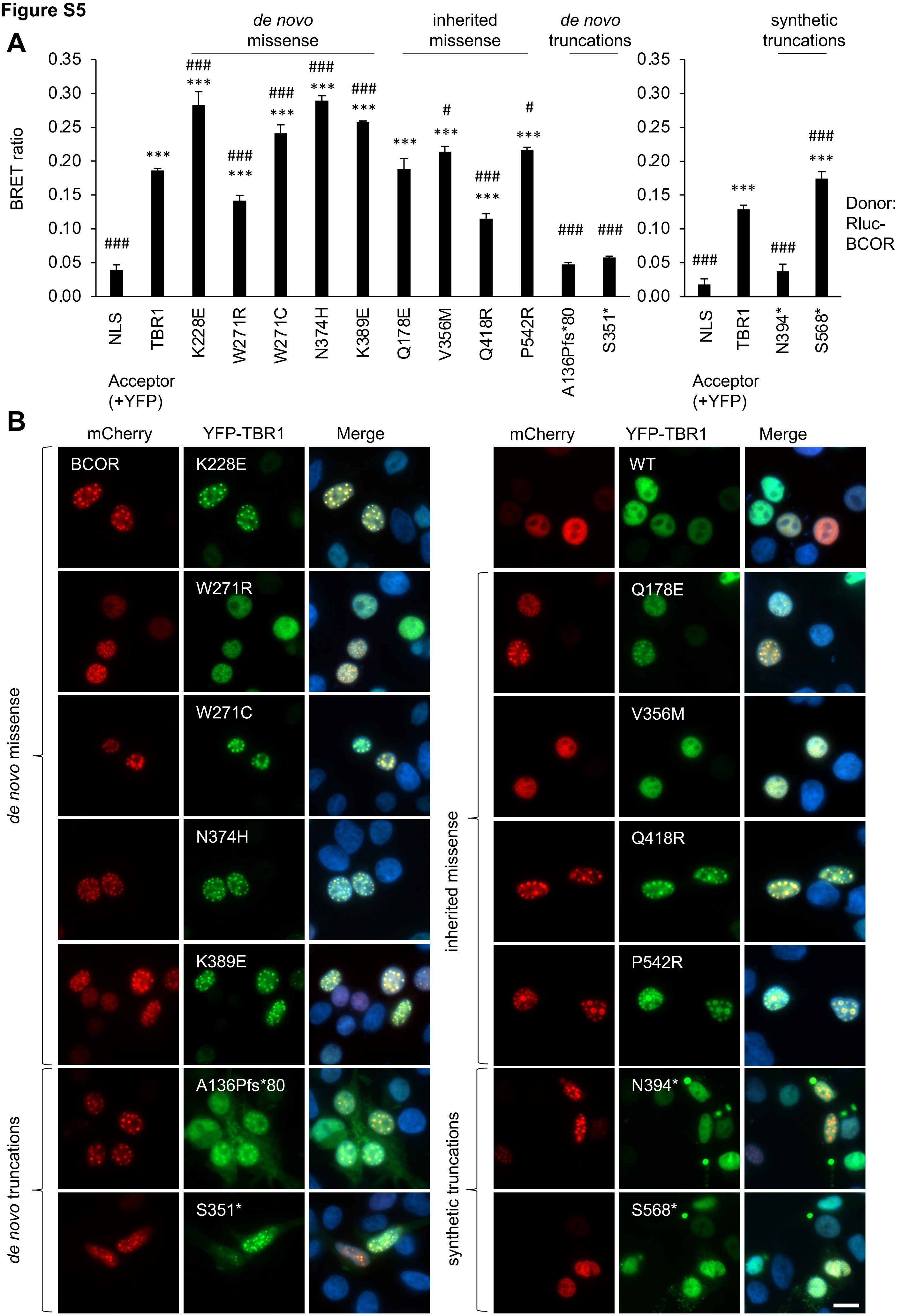
A
de novo missense
inherited missense
de novo truncations
synthetic truncations
BRET ratio
0.35
0.30
0.25
0.20
0.15
0.10
0.05
0.00
NLS
TBR1
K228E
W271R
W271C
N374H
K389E
Q178E
V356M
Q418R
P542R
A136Pfs*80
S351*
N394*
S568*
###
***
#
Acceptor (+YFP)
Donor: Rluc-BCOR
B
mCherry
YFP-TBR1
Merge
BCOR
WT
inherited missense
synthetic truncations
de novo truncations

Figure S6

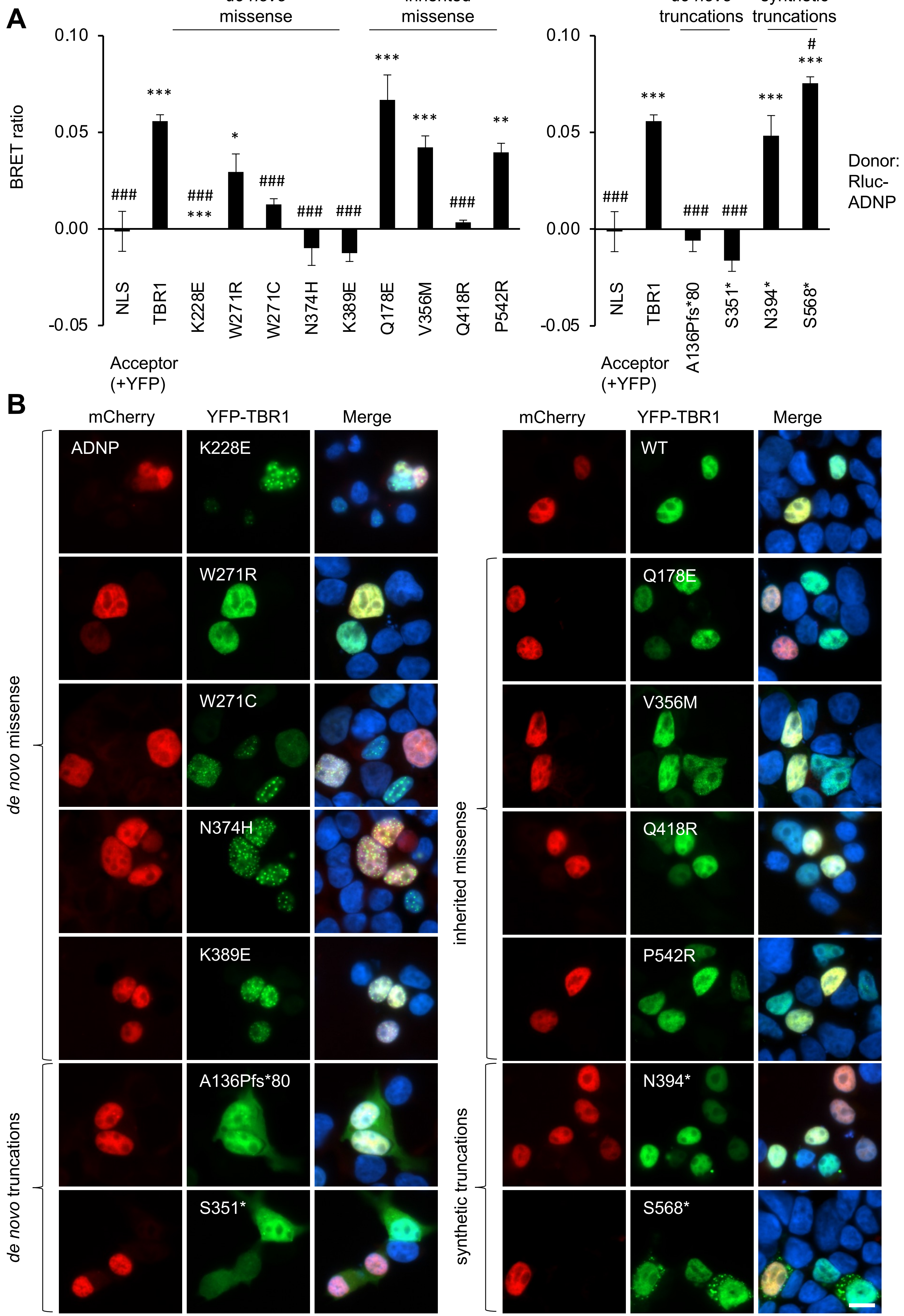

A
de novo missense
inherited missense
de novo truncations
synthetic truncations
BRET ratio
0.10
0.05
0.00
-0.05
###
***
###
***
*
###
###
###
***
***
###
**
###
***
###
###
***
#
***
NLS
TBR1
K228E
W271R
W271C
N374H
K389E
Q178E
V356M
Q418R
P542R
A136Pfs*80
S351*
N394*
S568*
Acceptor (+YFP)
Donor: Rluc-ADNP
B
mCherry
YFP-TBR1
Merge
ADNP
K228E
W271R
W271C
N374H
K389E
A136Pfs*80
S351*
WT
Q178E
V356M
Q418R
P542R
N394*
S568*
de novo missense
de novo truncations
inherited missense
synthetic truncations

Figure S7

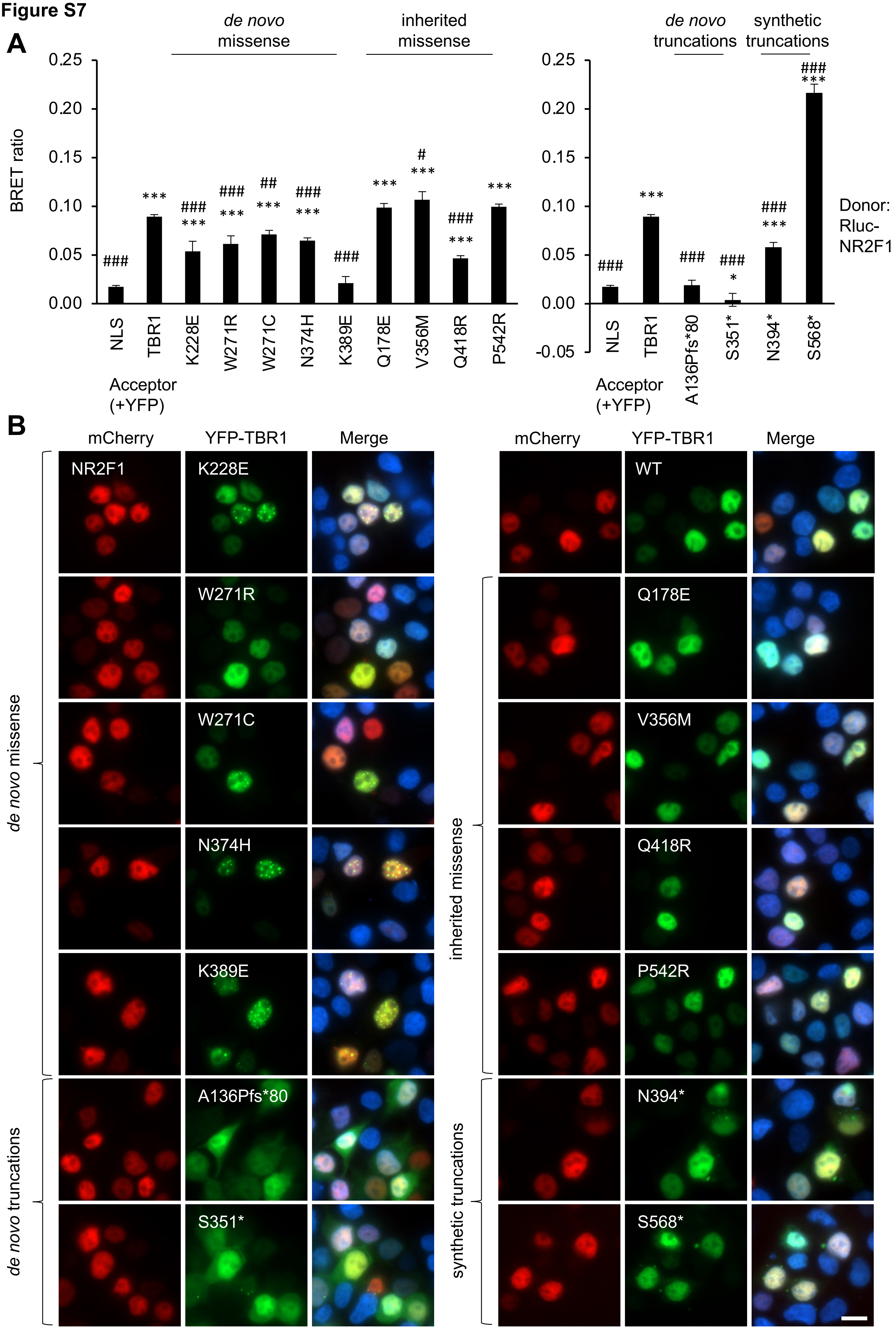

A
de novo missense
inherited missense
de novo truncations
synthetic truncations
BRET ratio
0.25
0.20
0.15
0.10
0.05
0.00
-0.05
###
***
##
#
*
NLS
TBR1
K228E
W271R
W271C
N374H
K389E
Q178E
V356M
Q418R
P542R
A136Pfs*80
S351*
N394*
S568*
Acceptor (+YFP)
Donor: Rluc-NR2F1
B
mCherry
YFP-TBR1
Merge
NR2F1
WT
de novo missense
inherited missense
de novo truncations
synthetic truncations

Figure S8

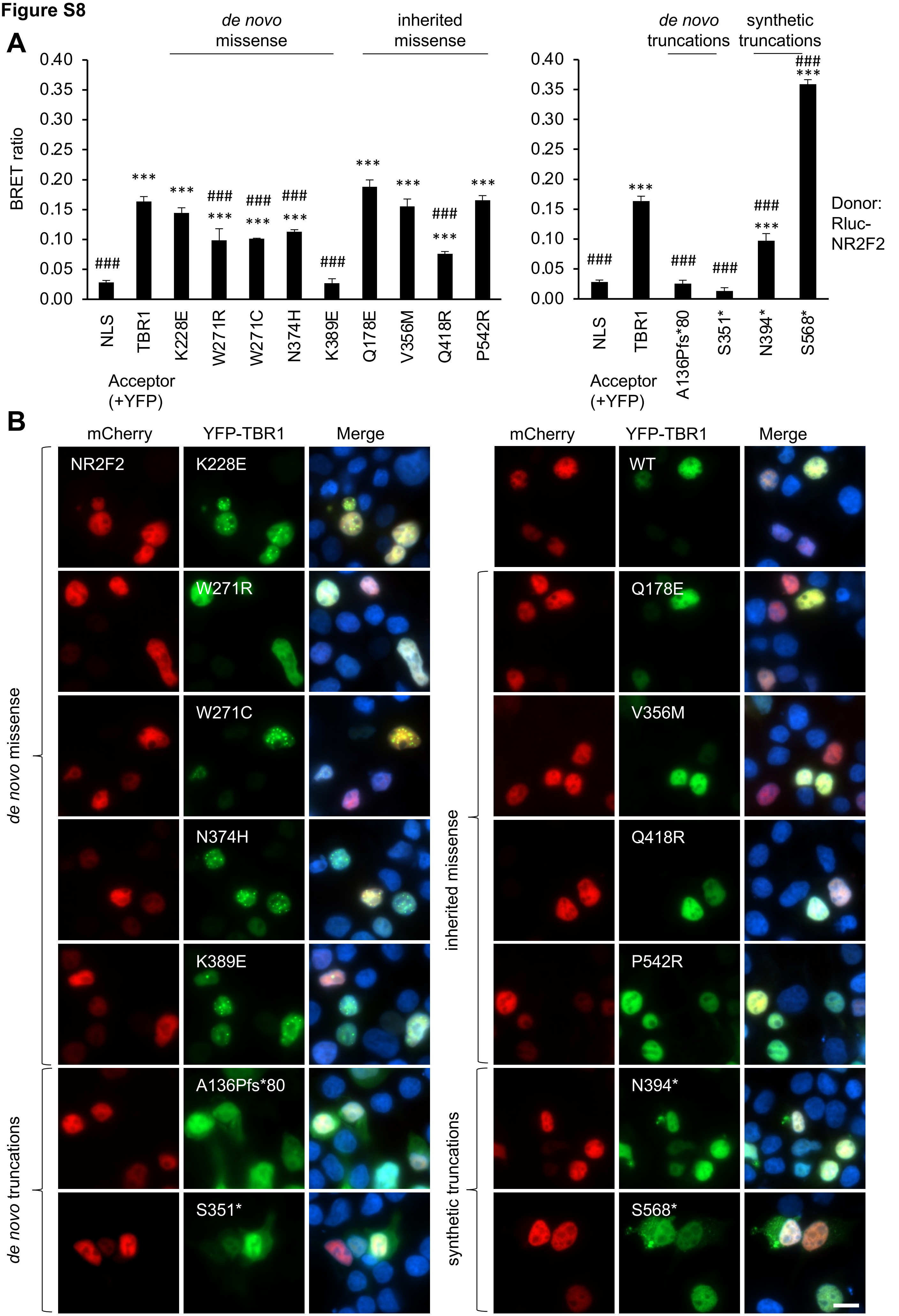

A
de novo missense
inherited missense
de novo truncations
synthetic truncations
BRET ratio
0.40
0.35
0.30
0.25
0.20
0.15
0.10
0.05
0.00
###
***
NLS
TBR1
K228E
W271R
W271C
N374H
K389E
Q178E
V356M
Q418R
P542R
A136Pfs*80
S351*
N394*
S568*
Acceptor (+YFP)
Donor: Rluc-NR2F2
B
mCherry
YFP-TBR1
Merge
NR2F2
WT
de novo missense
inherited missense
de novo truncations
synthetic truncations
